# Comprehensive Bibliometric Analysis of the Kynurenine Pathway in Mood Disorders: Focus on Gut Microbiota Research

**DOI:** 10.3389/fphar.2021.687757

**Published:** 2021-06-22

**Authors:** Xiuqing Zhu, Jinqing Hu, Shuhua Deng, Yaqian Tan, Chang Qiu, Ming Zhang, Xiaojia Ni, Haoyang Lu, Zhanzhang Wang, Lu Li, Yayan Luo, Shanqing Huang, Tao Xiao, Shujing Liu, Xiaolin Li, Dewei Shang, Yuguan Wen

**Affiliations:** ^1^ Department of Pharmacy, The Affiliated Brain Hospital of Guangzhou Medical University (Guangzhou Huiai Hospital), Guangzhou, China; ^2^ Guangdong Engineering Technology Research Center for Translational Medicine of Mental Disorders, Guangzhou, China; ^3^ Institute of Neuropsychiatry, The Affiliated Brain Hospital of Guangzhou Medical University (Guangzhou Huiai Hospital), Guangzhou, China

**Keywords:** kynurenine pathway, mood disorders, depression, bipolar disorder, bibliometric analysis, gut microbiota, microbiota-gut-brain axis, immune system

## Abstract

**Background:** Emerging evidence implicates the dysregulated kynurenine pathway (KP), an immune-inflammatory pathway, in the pathophysiology of mood disorders (MD), including depression and bipolar disorder characterized by a low-grade chronic pro-inflammatory state. The metabolites of the KP, an important part of the microbiota-gut-brain axis, serve as immune system modulators linking the gut microbiota (GM) with the host central nervous system.

**Aim:** This bibliometric analysis aimed to provide a first glimpse into the KP in MD, with a focus on GM research in this field, to guide future research and promote the development of this field.

**Methods:** Publications relating to the KP in MD between the years 2000 and 2020 were retrieved from the Scopus and Web of Science Core Collection (WoSCC), and analyzed in CiteSpace (5.7 R5W), biblioshiny (using R-Studio), and VOSviewer (1.6.16).

**Results:** In total, 1,064 and 948 documents were extracted from the Scopus and WoSCC databases, respectively. The publications have shown rapid growth since 2006, partly owing to the largest research hotspot appearing since then, “quinolinic acid.” All the top five most relevant journals were in the neuropsychiatry field, such as Brain Behavior and Immunity. The United States and Innsbruck Medical University were the most influential country and institute, respectively. Journal co-citation analysis showed a strong tendency toward co-citation of research in the psychiatry field. Reference co-citation analysis revealed that the top four most important research focuses were “kynurenine pathway,” “psychoneuroimmunology,” “indoleamine 2,3-dioxygenase,” and “proinflammatory cytokines,” and the most recent focus was “gut-brain axis,” thus indicating the role of the KP in bridging the GM and the host immune system, and together reflecting the field’s research foundations. Overlap analysis between the thematic map of keywords and the keyword burst analysis revealed that the topics “Alzheimer’s disease,” “prefrontal cortex,” and “acid,” were research frontiers.

**Conclusion:** This comprehensive bibliometric study provides an updated perspective on research associated with the KP in MD, with a focus on the current status of GM research in this field. This perspective may benefit researchers in choosing suitable journals and collaborators, and aid in the further understanding of the field’s hotspots and frontiers, thus facilitating future research.

## Introduction

Major depressive disorder (MDD) and depressive episodes of bipolar disorder (BD), which have similar clinical presentations, are both types of mood disorders (McIntyre et al., 2019; Vöhringer and Perlis, 2016). They are both associated with high suicide rates and can pose large burdens to societies and economies, according to the Global Burden of Disease study (GBD 2017 Disease and Injury Incidence and Prevalence Collaborators, 2018). Thus, understanding the molecular mechanisms of mood disorders is highly important for the development of effective treatment. Although the relationship between inflammation and mood disorders has been well documented, the complex pathophysiology of mood disorders has not been fully elucidated. Increasing preclinical and clinical studies provide evidence of alterations in the levels of pro-inflammatory and anti-inflammatory cytokines in MDD and other mood disorders (Chistyakov et al., 2018). Thus, mood disorders can be characterized by a low-grade chronic pro-inflammatory state, possibly owing to pathophysiological dysfunctions in immune-inflammatory pathways; this state might induce brain functional and structural alterations via multiple mechanisms (Squassina et al., 2019).

**FIGURE 1:**
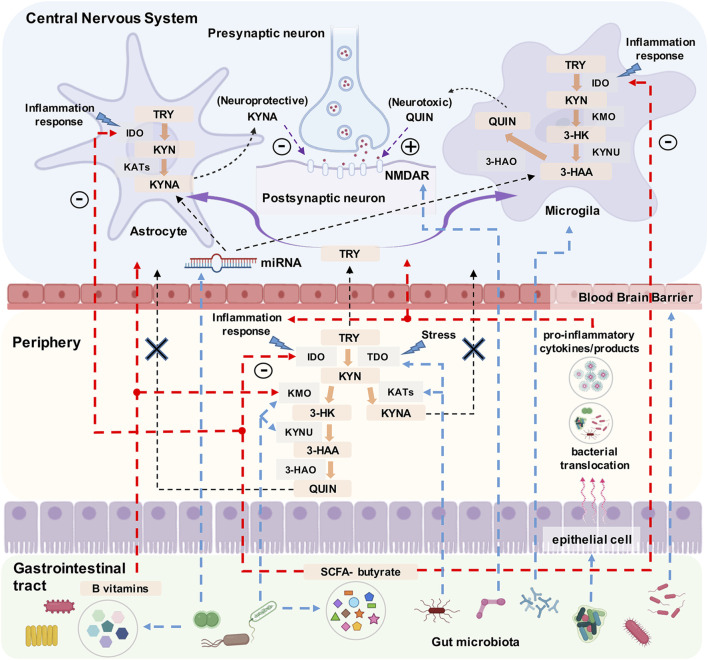
The balance between kynurenic acid (KYNA) and quinolinic acid (QUIN) in the central nervous system and the critical impact points (presented as blue dotted lines) on the kynurenine pathway (KP) under the control of the gut microbiota. Note. TRY: tryptophan, KYN: kynurenine, 3-HK: 3-hydroxykynurenine, KYNA: kynurenic acid, 3-HAA: 3-hydroxyanthranilic acid, QUIN: quinolinic acid, IDO: indoleamine 2,3-dioxygenase, TDO: tryptophan dioxygenase, KATs: kynurenine aminotransferases, KMO: kynurenine-3-monooxygenase, KYNU: kynureninase, 3-HAO: 3-hydroxyanthranlic acid oxygenase, NMDAR: N-methyl-d-aspartate receptor.

The kynurenine pathway (KP) is a potential inflammation-related mechanism implicated in the pathophysiology of mood disorders. Tryptophan (TRY) is an essential amino acid that serves as a precursor for serotonin (5-HT). However, kynurenine (KYN) is a major metabolite of TRY via the KP, which is responsible for 99% of dietary TRY metabolism (Russo et al., 2003; Won and Kim, 2016). The key primary enzymes in TRY oxidation metabolism via the KP are indoleamine 2,3-dioxygenase (IDO), which is expressed in all tissues, and tryptophan dioxygenase (TDO), which is mostly localized to the liver (Clarke et al., 2012; Kennedy et al., 2017). IDO and TDO expression are highly induced by the actions of inflammatory cytokines (particularly interferon-γ) and elevated levels of glucocorticoids in response to stress, respectively (Kennedy et al., 2017; Yoshida et al., 1981). Therefore, decreased levels of TRY may potentially influence serotonergic signaling in the brain, and IDO activity may also affect 5-HT biosynthesis (Zhang et al., 2016a). Downstream of IDO, KYN has two catabolic branches involving the formation of either kynurenic acid (KYNA) or quinolinic acid (QUIN) and intermediate metabolites, such as 3-hydroxykynurenine (3-HK) and 3-hydroxyanthranilic acid (3-HAA), which are collectively termed “kynurenines.” KYNA is a well-known N-methyl-d-aspartate (NMDA) receptor antagonist with antioxidant and neuroprotective effects, although its levels are diminished in inflammatory conditions (Lugo-Huitrón et al., 2011; Öztürk et al., 2020). In contrast, QUIN is an NMDA receptor agonist showing several neurotoxic effects, such as blocking uptake by astrocytes, whereas 3-HK is a neurotoxic compound that enhances oxidative stress and contributes to neurodegeneration, particularly in depression in late life (Leonard, 2018). The balance between KYNA and QUIN is shown in Figure 1. Therefore, if chronically imbalanced levels of neuroprotective and neurotoxic KP metabolites are not corrected, changes in the neuronal-glial network may result. This damage might be progressive and may make the brain more vulnerable to pathological conditions, thereby causing neuropsychiatric disorders, including depression and BD (Myint et al., 2007; Myint and Kim, 2014).

**FIGURE 2:**
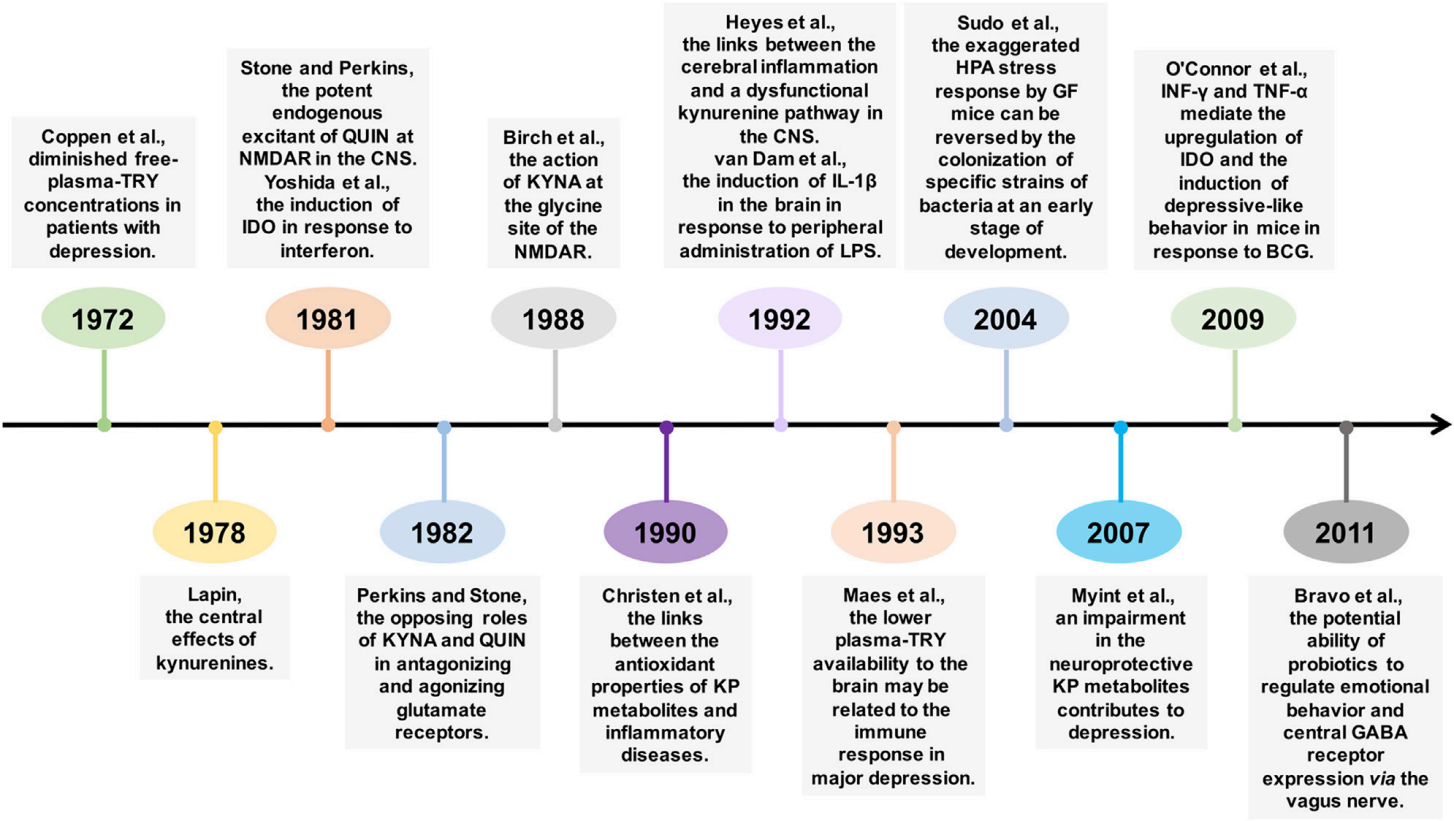
The timeline of key, seminal original discoveries that gave rise to the fields of research on the kynurenine pathway (KP) in mood disorders and the gut-brain axis. Note. TRY: tryptophan, QUIN: quinolinic acid, NMDAR: N-methyl-d-aspartate receptor, CNS: central nervous system, IDO: indoleamine 2,3-dioxygenase, KYNA: kynurenic acid, IL-1β: interleukin-1β, LPS: lipopolysaccharide, HPA: hypothalamic-pituitary-adrenal, GF: germ-free, GABA: gamma-aminobutyric acid, TNF-α: tumor necrosis factor-α, BCG: bacille Calmette-Guérin.

Figure 2 presents the timeline of key, seminal original discoveries that gave rise to the field of research on the KP in mood disorders and the gut-brain axis. For example, Coppen et al. (1972) reported diminished free-plasma-TRY concentrations in patients with depression, which may be a cause of low lumbar cerebrospinal fluid-TRY levels. Lapin. (1978) first discovered that intraventricularly injected QUIN produces seizures in mice, thus suggesting the central effects of kynurenines. Subsequently, Stone and Perkins. (1981) revealed that QUIN plays roles in neuronal excitation, possibly owing to its potent endogenous excitatory effects on NMDA receptors in the central nervous system (CNS). In the same year, Yoshida et al. (1981) first demonstrated that interferon induces IDO; thereafter, a study by Christen et al. (1990) suggested that the induction of IDO may stimulate a local antioxidant defense against inflammatory diseases, a response related to the antioxidant activities of some TRY metabolites in the KP. Subsequently, Perkins and Stone. (1982) discovered the opposing roles of KYNA and QUIN in antagonizing and agonizing glutamate receptors, respectively; the authors proposed that an imbalance in the KP might be associated with CNS disorders. Later, a study by Birch et al. (1988) first demonstrated that KYNA antagonizes NMDA responses via acting at the glycine site of the NMDA receptor. Notably, a recent review by Stone. (2020) concluded that there was overwhelming evidence for the action of QUIN on ionotropic glutamate receptors; conversely, no reliable or reproducible evidence for that on nicotinic receptors. A landmark study by Heyes et al. (1992) linked cerebral inflammation to a dysfunctional KP in the CNS. In the same year, van Dam et al. (1992) first demonstrated the induction of the expression of interleukin-1β in the brain in response to peripheral administration of lipopolysaccharide (LPS). Furthermore, Maes et al. (1993) proposed that the lower plasma-TRY availability to the brain may be related to the immune response in major depression. Direct clinical evidence from a study by Myint et al. (2007) provided support for the neurodegeneration hypothesis linking the imbalance between neuroprotective and neurodegenerative KP metabolites to the pathophysiology of major depression. In the gut microbiota field, an important study by Sudo et al. (2004) showed that germ-free mice have an exaggerated hypothalamic-pituitary-adrenal reaction to stress, as compared with the response in specific pathogen free mice, and this response could be reversed by the colonization of specific strains of bacteria at an early stage of development. O'Connor et al. (2009a) demonstrated an essential role of the pro-inflammatory cytokines interferon-γ and tumor necrosis factor-α on the induction of IDO and subsequent depressive-like behaviors in mice in the context of chronic inflammation induced by bacille Calmette-Guérin (an attenuated form of *Mycobacterium bovis*). Notably, the findings from Bravo et al. (2011) demonstrated the potential ability of probiotics to regulate emotional behavior and central gamma-aminobutyric acid receptor expression via the vagus nerve, thus highlighting the important role of bacteria in bidirectional gut-brain interactions. The further development of culture-independent molecular methods, such as 16S ribosomal RNA and metagenomic sequencing tools, has enabled researchers to better understand the structure and functions of the gut microbiome, thereby contributing to a key research hotspot, “gut microbiota,” occurring in 2011 (Zhu et al., 2021).

The microbiota-gut-brain axis is a bidirectional communication pathway linking the host central nervous system and gastrointestinal tract (Rhee et al., 2009; Hao et al., 2020), and one of the key communication pathways of the microbiota-gut-brain axis is the KP (Kennedy et al., 2017). The gut microbiota has multifocal effects on the KP, including synthesizing enzymes analogous to TDO, kynureninase, and kynurenine 3-monooxygenase; regulating both QUIN production, by influencing microglia cells, and NMDA receptor expression in the CNS; reducing the activities of multiple enzymes (including colon TRY hydrolase, IDO, and kynurenine aminotransferases); producing the short-chain fatty acid butyrate, which inhibits IDO transcription; and influencing the levels of cofactors (e.g., vitamins B_12_ and B_6_) of KP enzymes (Kennedy et al., 2017; Więdłocha et al., 2021). The gut microbiota can also affect the KP through epigenetic regulation of non-coding RNAs in the CNS. For example, Moloney et al. (2017) have demonstrated that the gut microbiota regulates the hippocampal microRNA-294-5p expression associated with KP metabolism. Normally peripheral KYNA and QUIN are not considered to easily cross the blood-brain barrier (BBB) (Kennedy et al., 2017); however, the gut microbiota can decrease the BBB permeability by up-regulating the expression of tight junction proteins (Braniste et al., 2014); thus, a permeable brain in germ-free animals can markedly increase the flow of peripheral KYNA and QUIN across the BBB (Więdłocha et al., 2021). The gut microbiota is also involved in the activation of epithelial cell gene expression through Toll-like receptors, thereby inducing pro-inflammatory cytokine production and release (Elson and Alexander, 2015), whereas only Toll-like receptor-3 stimulation has been reported to be associated with elevated KP metabolites (e.g., KYNA and QUIN) in human peripheral monocytes (Orhan et al., 2016). Greater gut dysbiosis can also lead to increased intestinal permeability, bacterial translocation, and the release of pro-inflammatory bacterial products into the circulatory system (Ferrucci and Fabbri, 2018). Subsequently, these pro-inflammatory cytokines and products may influence KP metabolism in both the peripheral and central nervous systems. The critical points in the KP under the control of the gut microbiota are shown in Figure 1.

In the past 2 decades, human and animal studies have increasingly been conducted to reveal the links between KP and mood disorders. Several meta-analyses have been reported (Arnone et al., 2018; Ogyu et al., 2018; Bartoli et al., 2020; Marx et al., 2020); however, these reviews have focused only on the relationship between the KP metabolites and mood disorders and included a relatively small number of articles. Additionally, although these meta-analyses have confirmed the abnormal levels of KP metabolites in mood disorders, further research on their exact etiological roles in mood disorders, particularly involving the microbiota-gut-brain axis, is required. To the best of our knowledge, the general aspects of the links between the KP and mood disorders and the current status of gut microbiota research in this field have not been systematically studied via bibliometric and visual analysis. Thus, researchers may have difficulty in attaining a comprehensive and macroscopic view of this field, because of the diverse research domains (Chen et al., 2020). Furthermore, additional innovations and breakthroughs might be hindered, owing to potential untimely analysis of research frontiers (Zhu et al., 2021). The present bibliometric analysis fills the gap in the literature in this field.

Bibliometric analysis is a statistical method used to rapidly quantitatively analyze and visualize scientific output, research hotspots, and developing trends, by using public literature databases (Zhang et al., 2020). This method has been applied to various disciplines including the medical sciences (Thompson and Walker, 2015). In the present study, we investigate the research output; discipline distribution; publication sources; and active countries/regions, institutions, and researchers, thus helping researchers choose suitable journals and collaborators. In addition, we combine journal co-citation analysis, reference co-citation analysis, keyword co-occurrence analysis, thematic map analysis, and keyword burst analysis to map the intellectual structure and to evaluate the research foundations, hotspots, and frontiers in the KP in mood disorders, with the aim of revealing the research status of gut microbiota in this field, guiding future research, and promoting the development of this field.

## Materials and Methods

### Data Sources and Search Strategy

**FIGURE 3:**
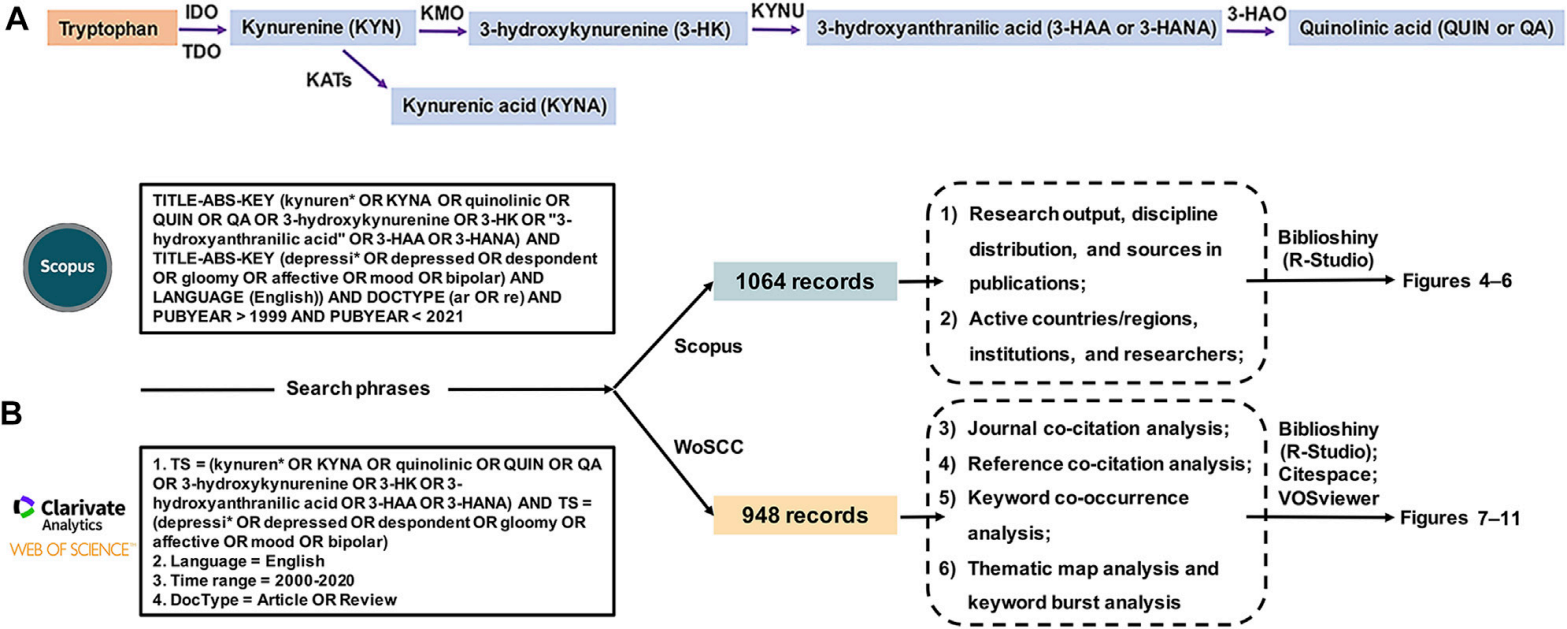
**(A)** The kynurenine pathway (KP) **(B)** The bibliometric analysis workflow, including detailed information on search phrases used in the Scopus and WoSCC databases, along with the corresponding numbers of extracted records, bibliometric analysis items, analysis tools, and generated figures. Note. IDO: indoleamine 2,3-dioxygenase, TDO: tryptophan dioxygenase, KATs: kynurenine aminotransferases, KYN: kynurenine, KYNA: kynurenic acid, KMO: kynurenine-3-monooxygenase, 3-HK: 3-hydroxykynurenine, KYNU: kynureninase, 3-HAA or 3-HANA: 3-hydroxyanthranilic acid, 3-HAO: 3-hydroxyanthranlic acid oxygenase, QUIN or QA: quinolinic acid.

A comprehensive search was conducted on two large, multidisciplinary citation databases, Scopus and Web of Science Core Collection (WoSCC) (Falagas et al., 2008), on a single day (January 1, 2021), to avoid the discrepancies due to daily database updates. These databases were chosen because Scopus is the world’s largest abstract and citation database (Zyoud et al., 2019), and WoSCC is a curated collection of high-quality scholarly peer-reviewed literature published worldwide (Zhu et al., 2021). The search phrases associated with the KP included KYN and its key metabolites (e.g., KYNA, QUIN, 3-HK, and 3-HAA) (Figure 3A), and the phrases associated with mood disorders included depression and related disorders such as BD. The search timespan covered the years 2000–2020, a period considered sufficiently long enough to reflect the development trends in this field that were our research focus. The present analysis was concerned with only two types of documents, articles and reviews published in the English language, and no species restrictions were imposed. All extracted records from both databases were identified after removal of duplicates. The same search strategy was applied to both databases, and detailed information is shown in Figure 3B.

### Data Analysis

The data from different databases were required to follow the fixed formats required by different bibliometric analysis tools. Given that converted data may be incompatible and affect subsequent analyses (Shi and Liu, 2019), the data from both databases were analyzed separately.

In the present study, the analyses of research output, discipline distribution, and sources in publications, and the active countries/regions, institutions, and researchers were conducted on the Scopus database by using the R-bibliometrix package (version 3.0.3, http://www.bibliometrix.org) in R-Studio (version 1.2.1335). Biblioshiny, a shiny app providing a web-interface for bibliometrix, was used to support the importation of metadata from databases and subsequent data management (Aria and Cuccurullo, 2017). The journal co-citation analysis, reference co-citation analysis, and keyword co-occurrence analysis were performed on the WoSCC database by using VOSviewer software (version 1.6.16, https://www.vosviewer.com/download), Citespace software (version 5.7 R5W, https://citespace.podia.com/courses/download), and the biblioshiny app, respectively. VOSviewer is a computer program developed by Nees Jan van Eck and Ludo Waltman from Leiden University (Leiden, the Netherlands) for bibliometric mapping. It can be used to conduct co-authorship analysis, keyword co-occurrence analysis, citation and co-citation analysis, and bibliographic coupling (van Eck and Waltman, 2010; Merigó et al., 2018). Citespace is a tool invented by Professor Chaomei Chen from Drexel University (Philadelphia, the United States of America) for conducting visualization analysis of scientific references. It is typically used to construct social network maps, co-citation network maps, and co-occurrence network maps (Chen, 2004; Chen, 2020). To obtain more convincing and reliable results, we performed an integrated analysis of thematic maps via the biblioshiny app and burst analysis of keywords via Citespace, on the basis of the WoSCC database, to examine the research frontiers in the field of the KP in mood disorders. A thematic map resulting from a clustering analysis of a co-occurrence network provides a Cartesian representation of the identified term clusters (Aria et al., 2020). It allows for easy visual interpretation of the research themes developed in a framework and provides information regarding emerging or declining themes (Aria and Cuccurullo, 2017). Similarly, burst analysis of keywords, which involves two attributes (i.e., the intensity and the duration of the burst), can reveal abrupt changes in keywords over a particular period, thus serving as an indicator of emerging research directions (Chen et al., 2014). The parameters used in the journal co-citation analysis via VOSviewer were as follows: counting method (fractional counting), minimum number of citations of a source (100), visualization weights (citations), normalization (association strength), clustering resolution (1.00), minimum cluster size (1), minimum line strength (200), and maximum lines (500). The parameters used in Citespace were as follows: time slicing (2000–2020), years per slice (1), term source (all selection), node type (one option chosen at a time from the reference in reference co-citation analysis, and the keyword in keyword co-occurrence analysis), selection criteria (top 40), pruning (none), and visualization (“cluster view-static” and “show merged network”). The bibliometric analysis items, as well as the corresponding databases and tools, and the figures in which the maps were visualized, are displayed in Figure 3B.

All statistical analyses were performed in IBM SPSS Statistics, version 25.0 (SPSS Inc., Chicago, IL, United States). Polynomial model fitting was used to predict the research output in 2021. The Spearman correlation coefficient was used to analyze the correlations between selected continuous variables. A *p*-value < 0.05 was considered statistically significant.

## Results

### Research Output, Discipline Distribution, and Sources in Publications

**FIGURE 4:**
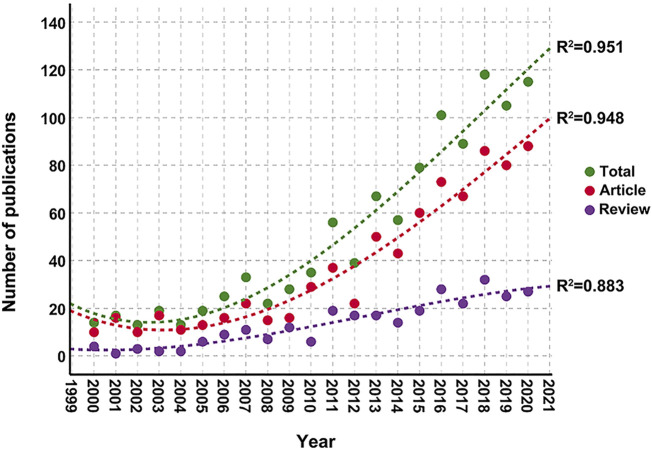
Annual trends in publication from Scopus (2000–2020) in the field of the kynurenine pathway (KP) in mood disorders and the corresponding polynomial fitted curves.

The total numbers of collected documents in the Scopus and WoSCC databases were 1,064 and 948, respectively, without duplications. Here, Scopus was selected as the data source of analysis, because of its better coverage of literature. Figure 4 shows that the total research output was very low before 2006, but the annual output of articles and reviews subsequently showed rapid upward trends. The annual growth rate calculated as (value in the year 2020/value in the year 2000) ^(1/20)^ −1, was 11.1%. A total of 781 articles and 283 reviews were retrieved from Scopus. Polynomial model fitting revealed significant correlations between the publication year and the publication output (the coefficients of determination (*r* ^2^) were 0.951, 0.948, and 0.883 for total documents, articles, and reviews, respectively). On the basis of polynomial curve fitting, the publication output is expected to reach approximately 130 in 2021, comprising 100 articles and 30 reviews.

Given that each document may involve several disciplines, the extracted studies mainly belonged to five disciplines: medicine (*n* = 516, 26.8%), neuroscience (*n* = 510, 26.5%), biochemistry, genetics and molecular biology (*n* = 293, 15.2%), pharmacology, toxicology and pharmaceutics (*n* = 230, 11.9%), and immunology and microbiology (*n* = 100, 5.2%). The literature included was published in 343 journals. There was a significant but weak positive relationship between the total publications (TP) of journals and their total citations (TC) (*r* = 0.593, *p* < 0.001). All the top five most relevant sources were in the field of neuropsychiatry and had impact factors (IFs) above 3: Brain Behavior and Immunity (IF _2019_ = 6.663, TP = 47, TC = 1,962), Psychoneuroendocrinology (IF _2019_ = 4.732, TP = 24, TC = 975), Progress in Neuro-Psychopharmacology and Biological Psychiatry (IF _2019_ = 4.361, TP = 19, TC = 1,064), Journal of Affective Disorders (IF _2019_ = 3.892, TP = 18, TC = 668), and Molecular Psychiatry (IF _2019_ = 12.384, TP = 18, TC = 2,368). Table 1 shows the top 20 journals that published the largest number of papers related to this field.

**TABLE 1:** The top 20 most relevant journals in the field of the kynurenine pathway (KP) in mood disorders between 2000 and 2020.

Rank	Source	Country	2019 JCR^®^ category (partition)	IF_2019_	TP	TC
1	Brain, Behavior, and Immunity	United States	Neurosciences (Q1), immunology (Q1), psychiatry (Q1)	6.633	47	1,962
2	Psychoneuroendocrinology	England	Neurosciences (Q1), psychiatry (Q1), endocrinology and metabolism (Q1)	4.732	24	975
3	Progress in Neuro-Psychopharmacology and Biological Psychiatry	England	Neurosciences (Q2), psychiatry (Q1), pharmacology and pharmacy (Q1), clinical neurology (Q1)	4.361	19	1,064
4	Journal of Affective Disorders	Netherlands	Psychiatry (Q1), clinical neurology (Q1)	3.892	18	668
5	Molecular Psychiatry	England	Neurosciences (Q1), psychiatry (Q1), biochemistry and molecular biology (Q1)	12.384	18	2,368
6	Journal of Neuroinflammation	England	Neurosciences (Q1), immunology (Q1)	5.793	17	1,095
7	PLoS One	United States	Multidisciplinary sciences (Q2)	2.740	16	400
8	Current Pharmaceutical Design	United Arab Emirates	Pharmacology and pharmacy (Q3)	2.208	15	621
9	Psychiatry Research	Netherlands	Psychiatry (Q3)	2.118	13	317
10	Frontiers in Psychiatry	Switzerland	Psychiatry (Q2)	2.849	12	163
11	Journal of Neural Transmission	Austria	Neurosciences (Q2), clinical neurology (Q2)	3.505	12	606
12	Journal of Neuroscience	United States	Neurosciences (Q1)	5.674	12	1,066
13	Neuroscience	England	Neurosciences (Q2)	3.056	12	752
14	Neuroscience and Biobehavioral Reviews	England	Neurosciences (Q1), behavioral sciences (Q1)	8.329	12	1,172
15	CNS and Neurological Disorders-Drug Targets	Netherlands	Neurosciences (N/A), pharmacology and pharmacy (N/A)	N/A	11	242
16	Journal of Psychiatric Research	England	Psychiatry (Q2)	3.745	11	1,237
17	Neuropsychopharmacology	England	Neurosciences (Q1), psychiatry (Q1), pharmacology and pharmacy (Q1)	6.751	11	1,541
18	Behavioral Brain Research	Netherlands	Neurosciences (Q3), behavioral sciences (Q2)	2.977	10	265
19	International Journal of Neuropsychopharmacology	England	Neurosciences (Q2), psychiatry (Q1), pharmacology and pharmacy (Q1), clinical neurology (Q1)	4.333	10	298
20	Neuropsychiatric disease and Treatment	New Zealand	Psychiatry (Q3), clinical neurology (Q3)	2.157	10	188

Note. JCR^®^: journal of citation reports in Web of Science, IF _2019_: impact factor in 2019, TP: total publications, TC: total citations, N/A: not available.

### Active Countries/Regions, Institutions, and Researchers

**FIGURE 5:**
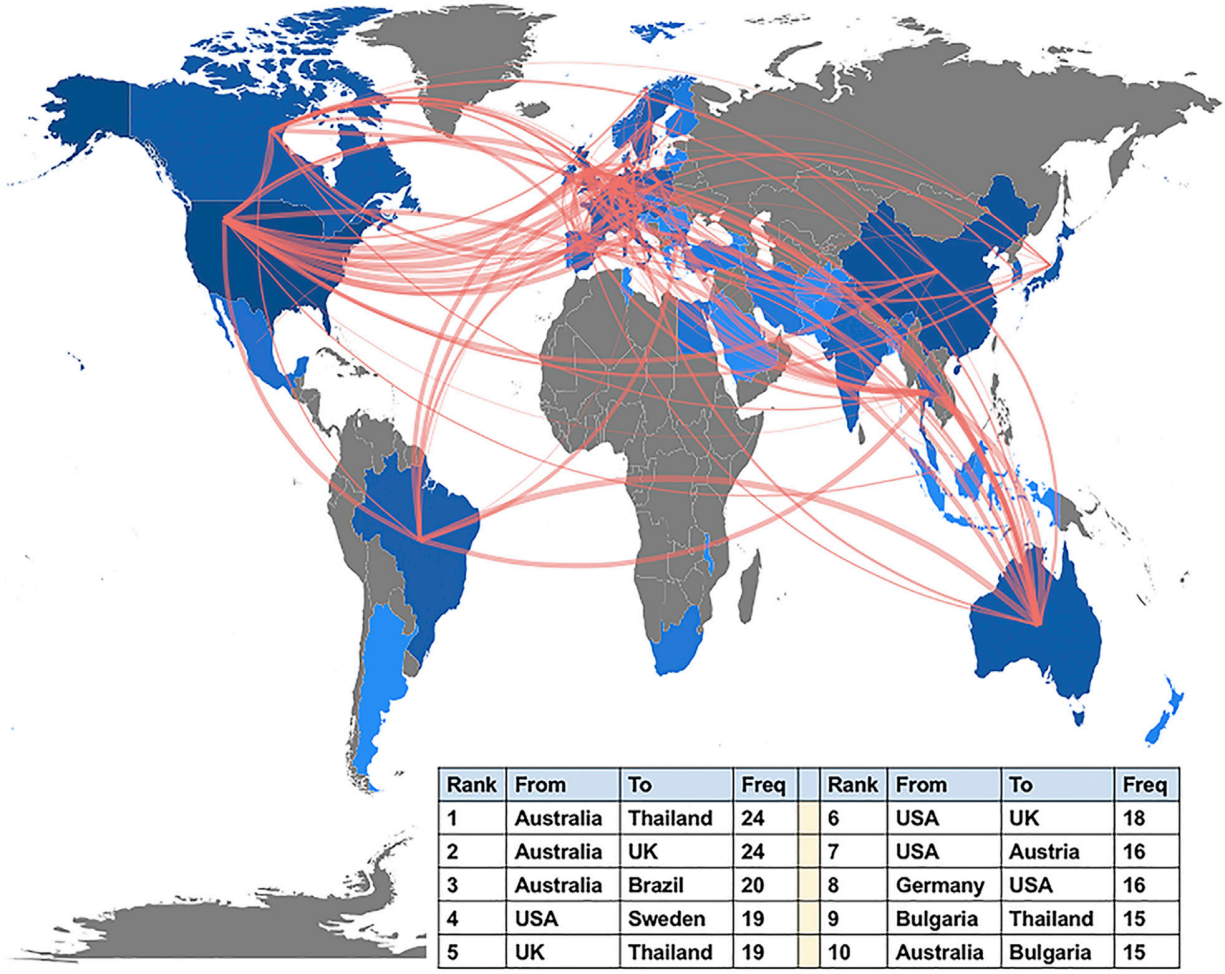
Country/region collaboration map of studies associated with the kynurenine pathway (KP) in mood disorders, generated through the biblioshiny app. Note. USA, United States; UK, United Kingdom.

The data extracted from Scopus indicated that the United States (TP = 871) was the most productive country, followed by China (TP = 319), Austria (TP = 220), Germany (TP = 213), and the United Kingdom (TP = 191). Figure 5 shows the country/region collaboration map worldwide generated by using the biblioshiny app. There were 280 pairs of collaborating countries/regions worldwide, of which the top three were Australia and Thailand with 24 collaborations, followed by Australia and United Kingdom with 24 collaborations, and Australia and Brazil with 20 collaborations.

**FIGURE 6:**
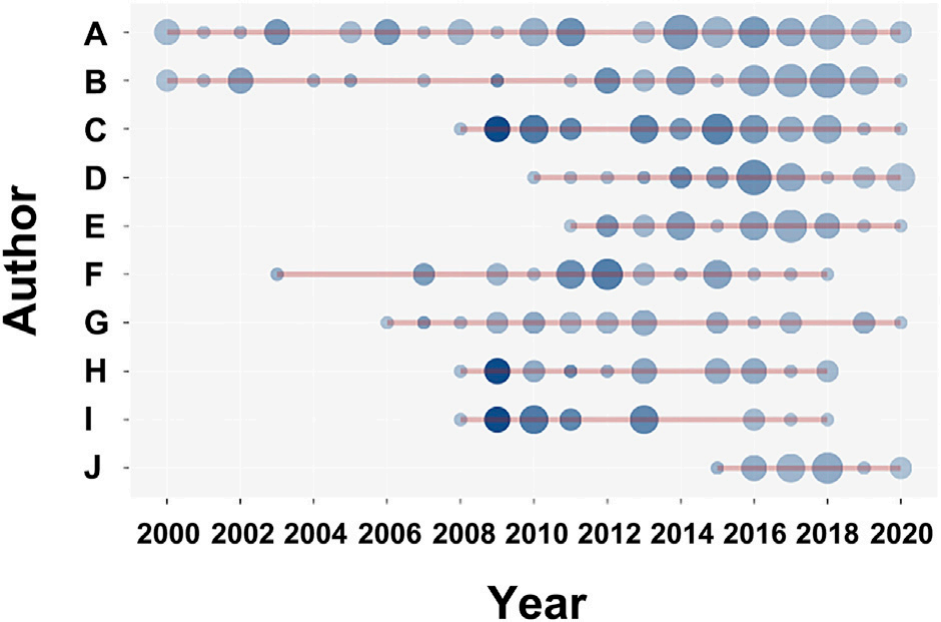
The top ten active researchers in the field of the kynurenine pathway (KP) in mood disorders and their publications over time. The larger the circle, the more articles published. The deeper the color, the more citations.

The most relevant affiliations were Innsbruck Medical University (TP = 90) in Austria, followed by the Karolinska Institutet (TP = 66) in Sweden, and the University of Illinois at Urbana-Champaign (TP = 54) in the United States. The most prolific researchers, whose names have been replaced with codes according to the principles of data protection, were Author “A” from Innsbruck Medical University (61 articles), followed by Author “B” from Chulalongkorn University in Thailand (44 publications), and Author “C” from the University of Texas, M. D. Anderson Cancer Center in the United States (34 publications). Table 2 and Figure 6 show the details of the top ten active researchers in this field and their productions over time, respectively.

**TABLE 2:** The top ten most prolific authors in the field of the kynurenine pathway (KP) in mood disorders between 2000 and 2020.

Rank	Author	Organization (Country/Region)	Major fields in publications	TP	TC	H-index
1	A	Medical university of innsbruck (Austria)	Psychiatry, neurosciences, endocrinology and metabolism	61	3,119	29
2	B	Chulalongkorn university (Thailand)/Medical university plovdiv (Bulgaria)/Deakin university (Australia)	Neurosciences, pharmacology and pharmacy, psychiatry	44	2,999	23
3	C	University of Texas dallas (United States)/UTMD anderson cancer center (United States)	Neurosciences, psychiatry, immunology	34	3,974	27
4	D	Karolinska institutet (Sweden)	Psychiatry, neurosciences, biochemistry and molecular biology	26	1,332	15
5	E	CRC scotland and london (England)/Clin res commun CRC scotland and london (England)	Neurosciences, pharmacology and pharmacy, clinical neurology	25	969	16
6	F	University of munich (Germany)	Psychiatry, neurosciences, clinical neurology	25	2,360	21
7	G	University of munich (Germany)	Psychiatry, neurosciences, clinical neurology	22	1,501	18
8	H	University of Texas health san antonio (United States)/Audie L. Murphy VA hospital (United States)	Neurosciences, psychiatry, immunology	20	2,611	18
9	I	University of Illinois system (United States)/Coll med (United States)	Neurosciences, immunology, psychiatry	18	3,045	16
10	J	University of toronto (Canada)/Center for addiction and mental health (Canada)/Deakin university (Australia)	Neurosciences, psychiatry, pharmacology and pharmacy	16	331	10

Note. TP: total publications, TC: total citations.

### Journal Co-citation Analysis

**FIGURE 7:**
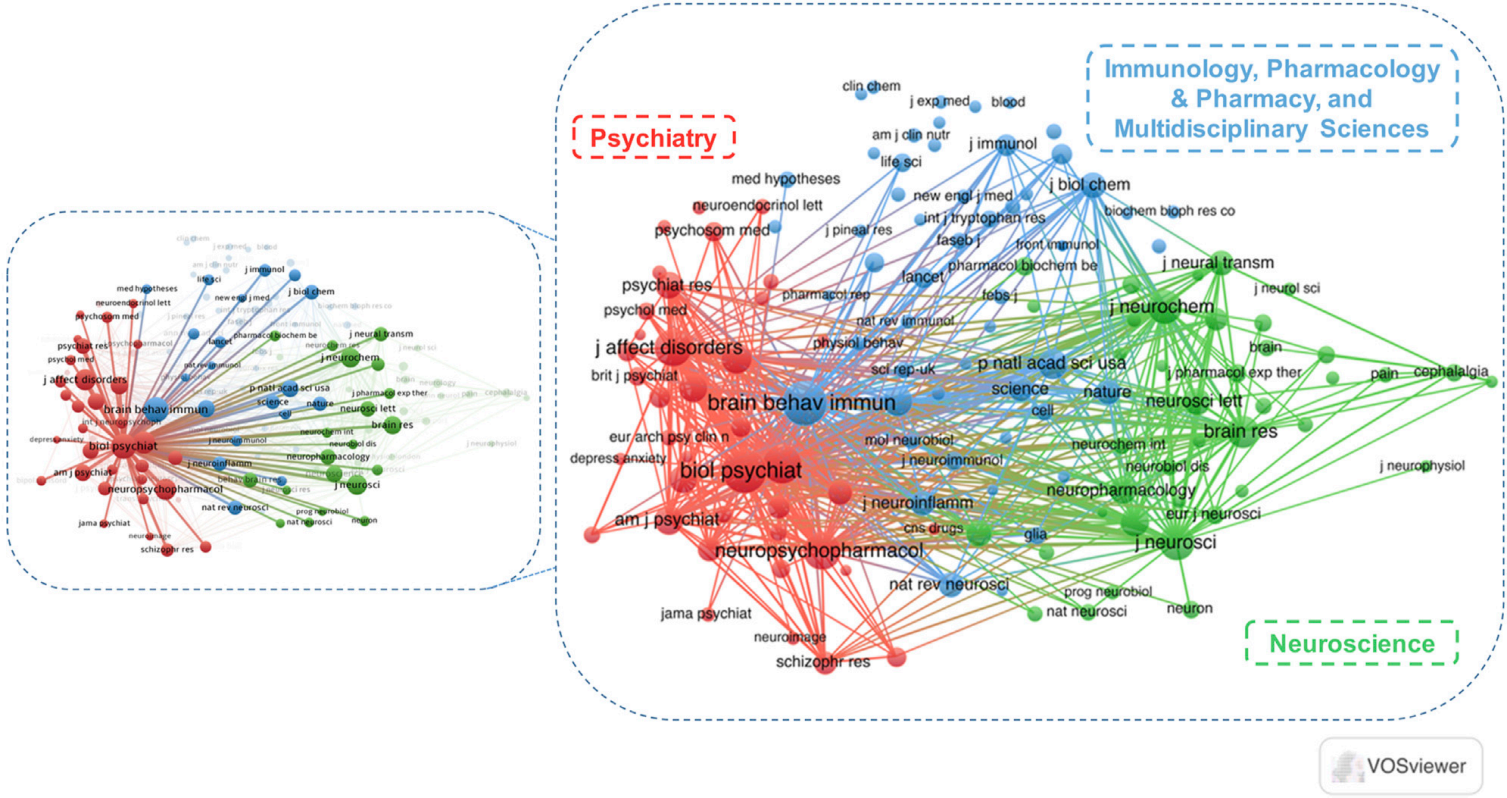
Cluster visualization of the journal co-citation analysis. Each node represents a journal, and the size of each circle is determined by the co-citations of the journal.

**FIGURE 8:**
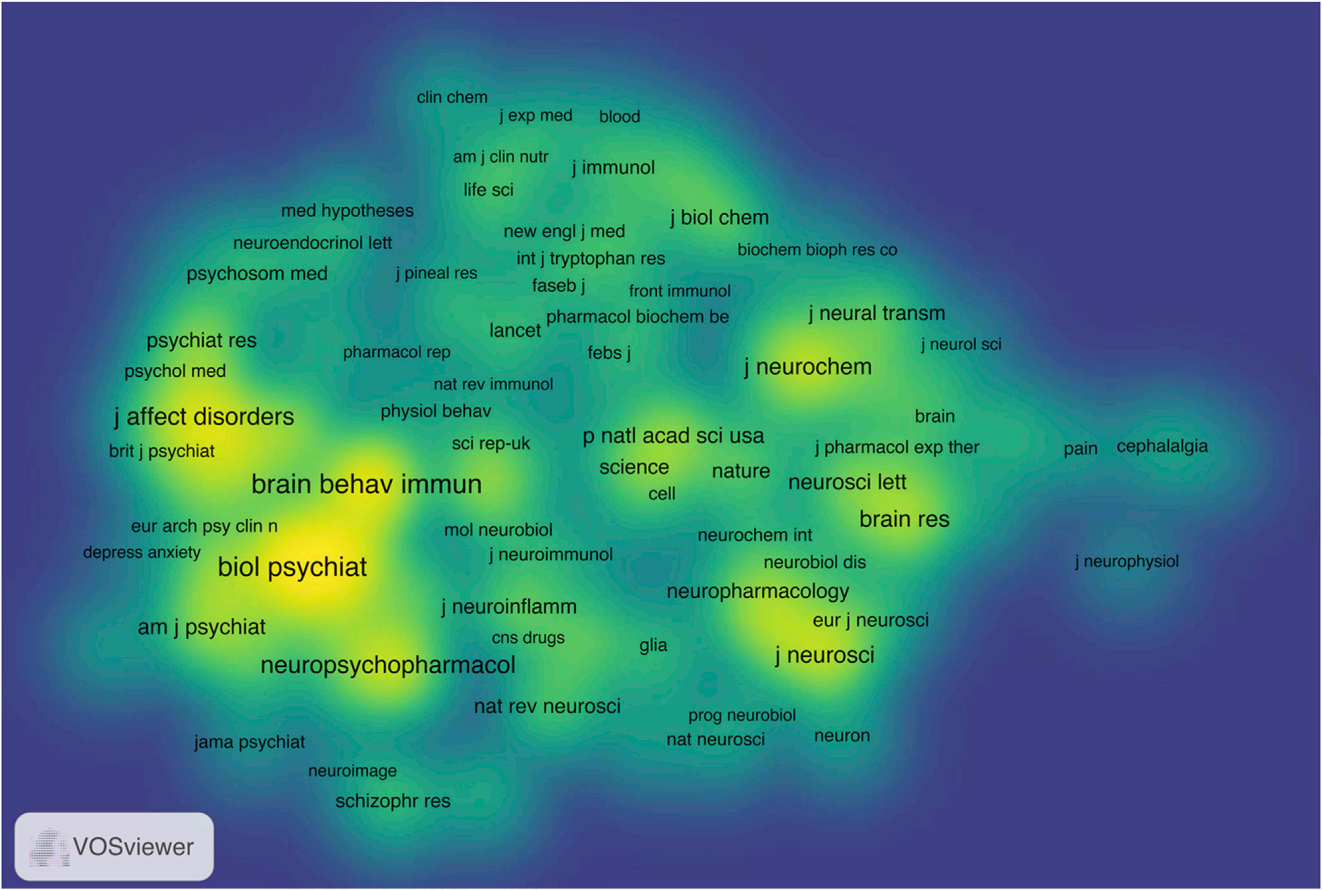
Item density visualization of the journal co-citation analysis. The larger the number of the neighboring items and the higher the weights of the items in a point, the closer the color of the point is to yellow in the visualization.

Journal co-citation analysis, first introduced by McCain (1991), focuses primarily on the journal-to-journal relationship and is widely used to study the interdisciplinary structure of a given academic field (Chi and Young, 2013; Hsiao and Yang, 2011; Yang et al., 2019). The network map yielded co-citation patterns of 132 journals, which were divided into three clusters, “Psychiatry (red cluster),” “Immunology, Pharmacology and Pharmacy, and Multidisciplinary Sciences (blue cluster),” and “Neuroscience (green cluster),” via manual assignment of their descriptive labels based on the subject categories (Figure 7) (Najas-Garcia et al., 2018). The easily interpreted visualization of item density indicates a strong tendency toward the co-citation of journals in these dense areas, including the cluster “Psychiatry” (Figure 8) (van Eck and Waltman, 2010). Highly co-cited journals are those that are frequently cited together by other journals; in these journals, the published articles reflect the important research fundaments of the field (Guo et al., 2019). The top two journals on the basis of total co-citations (TCC) were Biological Psychiatry (IF _2019_ = 12.095, TCC = 2009, total link strength = 1876.75), and Brain Behavior and Immunity (IF _2019_ = 6.663, TCC = 1923, total link strength = 1787.65). Figure 7 shows a strong tendency toward co-citation relationships between the journal Biological Psychiatry and other journals from these three clusters.

### Reference Co-citation Analysis

**FIGURE 9:**
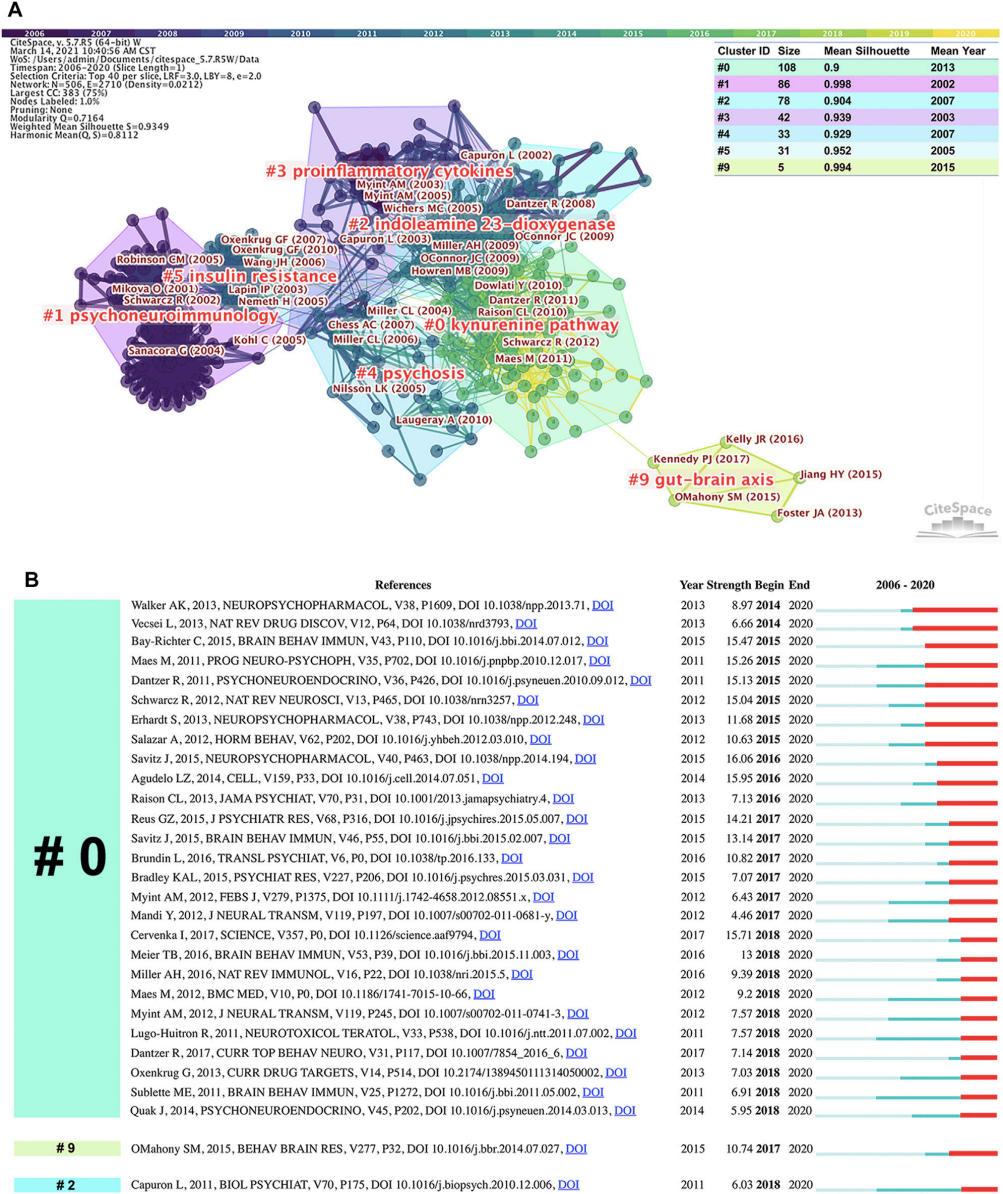
| **(A)** Cluster visualization of the co-citation network of references via Citespace, together with the details and the top five representative references of the generated clusters **(B)** The references with the strongest currently ongoing citation bursts in this field. The red bars indicate the duration of the burst and reflect references cited frequently; the green bars indicate references cited infrequently.

Highly co-cited references are those that are frequently cited together by other articles, and thus, can be regarded as knowledge bases in a particular field. In this section, the WoSCC database was used as the data source for analysis because of its coverage of high-quality literature. Figure 9A presents the largest seven clusters of the co-citation network of references, together with their details and the top five representative references, on the basis of a log-likelihood ratio algorithm in Citespace software: “kynurenine pathway (cluster #0),” “psychoneuroimmunology (cluster #1),” “indoleamine 2,3-dioxygenase (cluster #2),” “proinflammatory cytokines (cluster #3),” “psychosis (cluster #4),” “insulin resistance (cluster #5),” and “gut-brain axis (cluster #9).” All clusters were constructed on the basis of keywords extracted from the references. The total modularity Q-value of 0.7164 and the mean silhouette of each cluster above 0.7 indicated that the clustering structure was significant, and the results were highly credible (Guo et al., 2019). The burst detection of references can reveal the abrupt changes in citations over time, thereby indicating the evolution of a knowledge domain (Synnestvedt et al., 2005). Figure 9B shows the references with the strongest citation bursts that are currently ongoing, most of which belonged to cluster #0, labeled “kynurenine pathway.”

Table 3 shows the top ten co-cited references (four reviews, five articles, and a meta-analysis) in this field, which were found mostly in clusters #0 and #2. The review published in Nature Reviews Neurosciences by Schwarcz et al. (2012), from the University of Maryland, United States, was the most co-cited reference (TCC = 163). Table 4 presents the representative references in cluster #9, labeled “gut-brain axis,” in which the review published in Behavioral Brain Research by O'Mahony et al. (2015) had the strongest currently ongoing citation bursts (Figure 9B). Highly co-cited references are commonly important foundational studies that contribute to future researchers’ understanding of the research foundations in the field.

**TABLE 3:** The top ten co-cited references related to the kynurenine pathway (KP) in mood disorders between 2000 and 2020.

Rank	References	Title	Source	Type	Total co-citations	In cluster
1	Schwarcz et al. (2012)	Kynurenines in the mammalian brain: When physiology meets pathology	Nature Reviews Neuroscience	Review	163	#0
2	Raison et al. (2010)	CSF concentrations of brain tryptophan and kynurenines during immune stimulation with IFN-alpha: Relationship to CNS immune responses and depression	Molecular Psychiatry	Article	127	#0
3	O'Connor et al. (2009b)	Lipopolysaccharide-induced depressive-like behavior is mediated by indoleamine 2,3-dioxygenase activation in mice	Molecular Psychiatry	Article	117	#2
4	Dantzer et al. (2008)	From inflammation to sickness and depression: When the immune system subjugates the brain	Nature Reviews Neuroscience	Review	108	#2
5	Dantzer et al. (2011)	Inflammation-associated depression: From serotonin to kynurenine	Psychoneuroendocrinology	Review	96	#0
6	Maes et al. (2011)	The new '5-HT′ hypothesis of depression: Cell-mediated immune activation induces indoleamine 2,3-dioxygenase, which leads to lower plasma tryptophan and an increased synthesis of detrimental tryptophan catabolites (TRYCATs), both of which contribute to the onset of depression	Progress in Neuro-Psychopharmacology and Biological Psychiatry	Review	94	#0
7	Dowlati et al. (2010)	A meta-analysis of cytokines in major depression	Biological Psychiatry	Meta-analysis	89	#0
8	Steiner et al. (2011)	Severe depression is associated with increased microglial quinolinic acid in subregions of the anterior cingulate gyrus: Evidence for an immune-modulated glutamatergic neurotransmission?	Journal of Neuroinflammation	Article	86	#0
9	Myint et al. (2007)	Kynurenine pathway in major depression: Evidence of impaired neuroprotection	Journal of Affective Disorders	Article	83	#0
10	Bay-Richter et al. (2015)	A role for inflammatory metabolites as modulators of the glutamate N-methyl-d-aspartate receptor in depression and suicidality	Brain, Behavior, and Immunity	Article	83	#0

**TABLE 4:** The top five representative references of cluster #9, labeled “gut-brain axis” in the co-citation network of references.

Rank	References	Title	Source	Type	Total co-citations
1	O'Mahony et al. (2015)	Serotonin, tryptophan metabolism and the brain-gut-microbiome axis	Behavioral Brain Research	Review	23
2	Kennedy et al. (2017)	Kynurenine pathway metabolism and the microbiota-gut-brain axis	Neuropharmacology	Review	6
3	Foster and McVey Neufeld (2013)	Gut-brain axis: how the microbiome influences anxiety and depression	Trends in Neurosciences	Review	6
4	Jiang et al. (2015)	Altered fecal microbiota composition in patients with major depressive disorder	Brain, Behavior, and Immunity	Article	5
5	Kelly et al. (2016)	Transferring the blues: Depression-associated gut microbiota induces neurobehavioural changes in the rat	Journal of Psychiatric Research	Article	5

### Keyword Co-Ocurrence Analysis

Keyword co-occurrence analysis, aiming to investigate the co-occurring relationships between keywords in a set of publications, can reflect hot topics and help researchers deepen their understanding of scientific findings in research hotspots. “Keywords Plus” comes from a glossary defined by the Thomson Reuters editorial expertize team. This state-of-the-art keyword searching tool has been confirmed to be more broadly descriptive than the “Author Keywords,” thus enhancing the power of cited-reference searching (Zhang et al., 2016b).

**FIGURE 10:**
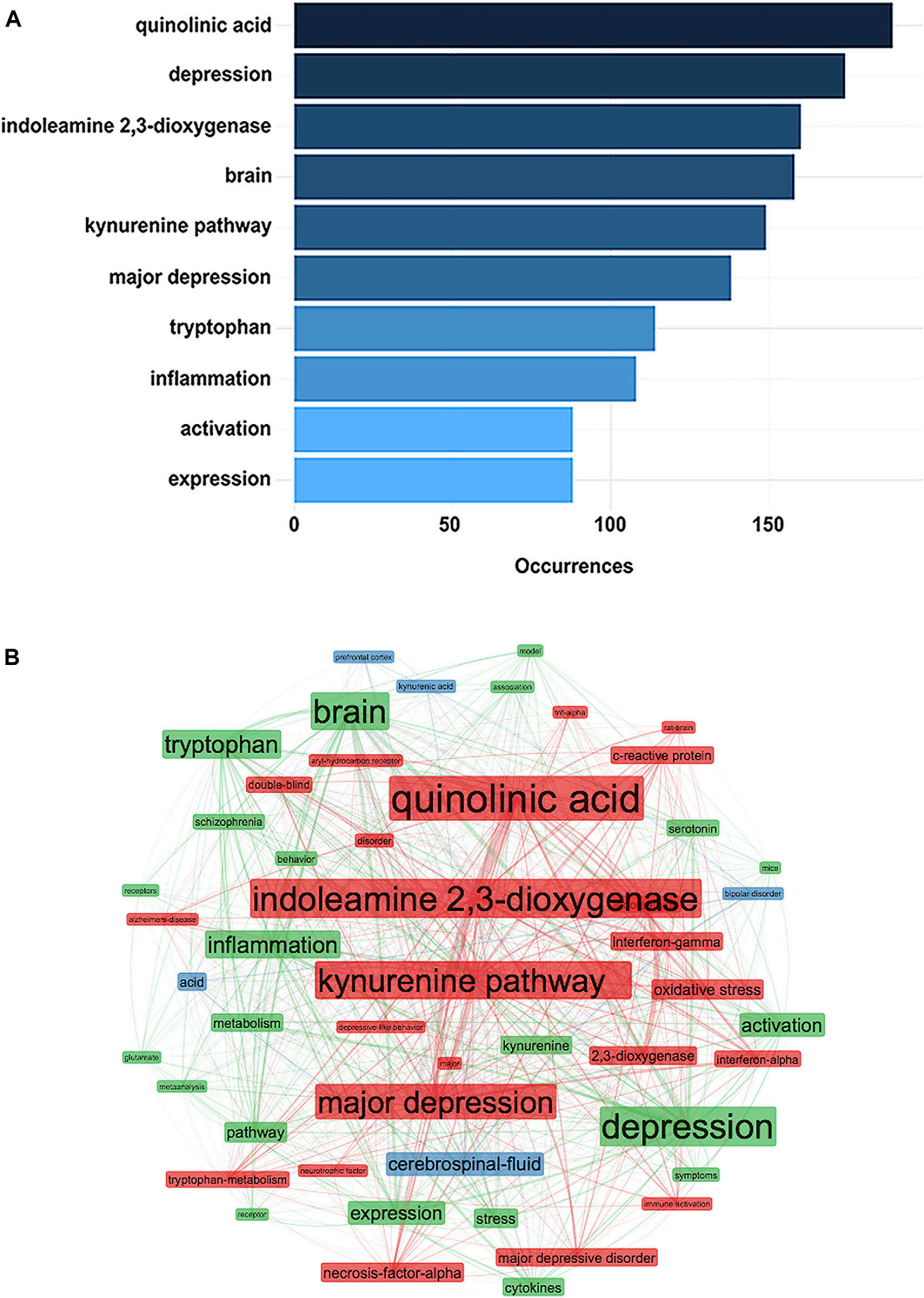
| **(A)** The top ten highest frequency keywords extracted by using “Keywords Plus” from the WoSCC database via the biblioshiny app **(B)** Cluster visualization of the keyword co-occurrence network among the 50 main keywords via the biblioshiny app, presented in a sphere network layout.

Figure 10A presents the top ten highest frequency keywords, on the basis of the WoSCC database, by using the “Keywords Plus” parameter via the biblioshiny app. The term “quinolinic acid” occurred in 2006 and ranked first with 189 occurrences, followed by “depression” with 174 occurrences, and “indoleamine 2,3-dioxygenase” with 160 occurrences. Figure 10B shows the keyword co-occurrence network among the 50 main keywords, by using the “Keywords Plus” parameter and the “Louvain” clustering algorithm via the biblioshiny app, in which three clusters with different colors (red, green, and blue) were established. The most relevant nodes belonged to the same clusters with the same colors, which represented close co-occurring relationships. The node size and link line width were proportional to the extent of co-occurrence and the strength of co-occurring relationships between nodes, respectively (Rodríguez-Sabiote et al., 2020). Additionally, the betweenness centrality was used to measure the importance of nodes in the network. The more important the node, the higher the betweenness centrality, thus indicating that more information passed through the node (Rodríguez-Sabiote et al., 2020). The hot topic “quinolinic acid,” which belonged to the red cluster, notably was the largest and had the highest calculated betweenness centrality value of 57.01.

### Thematic Map Analysis and Burst Analysis of Keywords

In the present study, the terms from “Keywords Plus,” according to the WoSCC database, could be considered emerging topics within this field. Given that differences might exist in analysis results across bibliometric tools, an overlap analysis between the thematic map of keywords from the biblioshiny app and the keyword burst analysis from Citespace software was conducted to identify research frontiers in this field.

**FIGURE 11:**
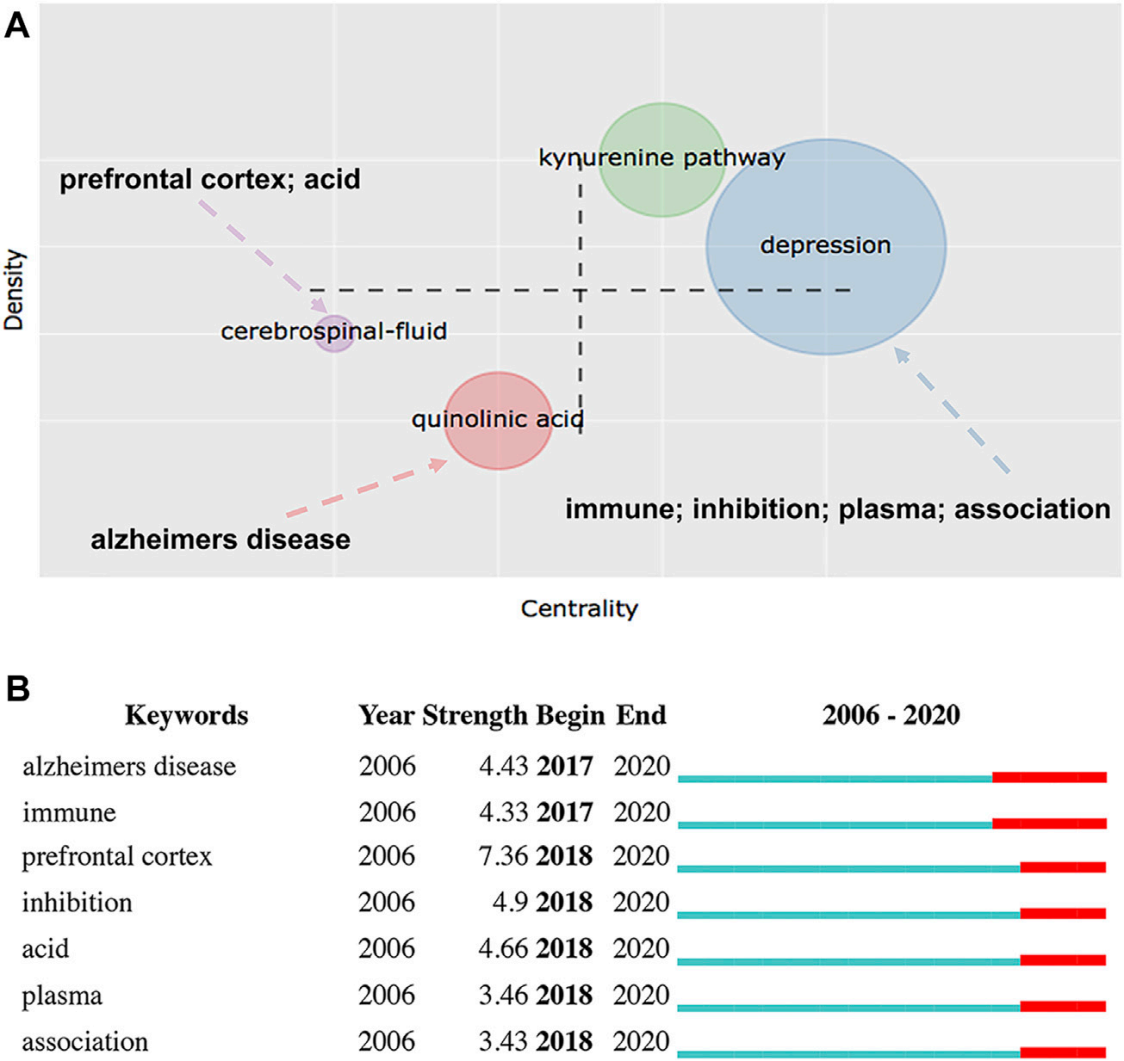
| **(A)** Thematic map of “Keywords Plus” created by the biblioshiny app **(B)** Keywords with the strongest currently ongoing citation bursts. The red bars indicate the duration of the burst and reflect keywords cited frequently; in contrast, the green bars indicate keywords cited infrequently. These keywords identified by Citespace were found within the clusters labeled “depression,” “quinolinic acid,” and “cerebrospinal-fluid” in the thematic map.

The thematic map was created by using a two-dimensional matrix involving two types of measurement: centrality and density. Figure 11A shows the thematic map of “Keywords Plus,” in which the *X*-axis indicates the centrality, i.e., the importance of a theme, and the *Y*-axis represents the density, a measure of the development of a theme (Aria et al., 2020). Accordingly, the upper right quadrant (i.e., quadrant 1) pertains to motor themes that are both important and well-developed, the upper left quadrant (i.e., quadrant 2) is associated with highly developed and isolated themes, the lower left quadrant (i.e., quadrant 3) refers to emerging or declining themes, and the lower right quadrant (i.e., quadrant 4) contains transversal and basic themes (Cobo et al., 2011). The clusters are represented by bubbles within the map, which are labeled by the keywords with the highest occurrences, and their sizes are proportional to the keyword occurrences (Rodríguez-Sabiote et al., 2020). Notably, two clusters corresponding to “quinolinic acid” and “cerebrospinal-fluid,” composed of 43 and 44 keywords, respectively, were positioned in quadrant 3, which was characterized by both low centrality and low density.

According to keyword burst analysis, the top 47 keywords were found to have the strongest citation bursts during the period from 2006 to 2020. Figure 11B shows the meaningful keywords with the strongest citation bursts that are currently ongoing, including “Alzheimer’s disease,” “immune,” “prefrontal cortex,” “inhibition,” “acid,” “plasma,” and “association.” An overlap analysis indicated that only three terms, “Alzheimer’s disease,” “prefrontal cortex,” and “acid,” were found within the clusters in quadrant three of Figure 11A. Among them, the term “prefrontal cortex,” appearing in 2018, had the highest strength, thus reflecting its highest frequency of citation. Hence, these three terms were identified as research frontiers in this field.

## Discussion

To date, kynurenines have been implicated in a variety of diseases, particularly psychiatric disorders, owing to their neuromodulatory properties (Chen and Guillemin, 2009; Savitz, 2020). The roles of variations in the levels of kynurenines in the pathogenesis of mood disorders have gained increasing attention, thus making the KP a hotspot in the field of mood disorders in the past 2 decades. Currently, bibliometric analysis is increasingly being used to review the status and trends in a particular field (Zhang et al., 2020). However, to date, no bibliometric analyses focusing on the KP in mood disorders, particularly the gut microbiota research in this field, have been reported.

The current analysis showed that the number of publications on the KP in mood disorders increased rapidly since 2006, a result possibly associated with the many active researchers in different disciplines (e.g., Authors “A–F”) with interests within this field, and several hotspots (e.g., QUIN) having occurred since then. Most of the top relevant journals had high IFs. Moreover, a weak positive relationship between the TP and TC of journals in this field indicated that the research activity regarding the KP in mood disorders in general was favored by high-IF journals in the field of neuropsychiatry. In addition, our journal co-citation analysis showed a strong tendency toward co-citation of research in the field of psychiatry, and the journal Biological Psychiatry received many co-citations despite its few publications related to this research area, thus indicating that the studies in this journal served as important research foundations in this field.

Among countries, the United States had an absolute advantage in the research output in this field, followed by China. Remarkably, active scientific collaborations between the United States and countries such as Sweden, United Kingdom, and Austria, were found. Although China, a developing country, has shown extensive development in this field, its influence was relatively low, owing to insufficient cooperative relationships with the most prolific countries; thus, more international scientific cooperation is needed. Austria was the third most productive country, possibly because Author “A,” the most active scholar in this field, is from Innsbruck Medical University, which is also a highly relevant affiliation in this field. Interestingly, active collaborative relationships between Thailand and countries such as Australia, the United Kingdom, and Bulgaria were also observed and were likely to be associated with the influential expert, Author “B” from Chulalongkorn University, who has a history of close long-term cooperation with researchers (e.g., Authors “E” and “J”) from Deakin University (Australia), CRC Scotland and London (United Kingdom), Medical University of Plovdiv (Bulgaria), Federal University of Ceará (Brazil), and University of Toronto (Canada). Among the top ten active researchers in the field of the KP in mood disorders, Authors “B,” “E,” and “J” were also among the top ten most prolific authors in the gut microbiota research in the depression field, according to our previous published bibliometric analysis (Zhu et al., 2021).

In the current analysis of the reference co-citation network, the presence of the largest cluster #0, “kynurenine pathway,” occurring in 2013, was unsurprizing, because this enzymatic cascade involves a group of related-compounds, the kynurenines, originating from TRY. The next three largest clusters were “psychoneuroimmunology,” “indoleamine 2,3-dioxygenase,” and “proinflammatory cytokines,” and the most recent one was “gut-brain axis,” thus indicating the role of KP in bridging the gut microbiota and the host immune system. Most of the top ten co-cited references belonged to cluster #0, “kynurenine pathway,” thus identifying important knowledge foundations in this field. For example, the most influential review, published by Schwarcz et al. (2012), and entitled “Kynurenines in the mammalian brain: when physiology meets pathology,” was co-cited more than 160 times. This review describes the metabolism and regulation of neuro-active kynurenines in the brain, and the communication pathways linking the peripheral and central KP, then explains how the dysregulation of the KP is associated with neurological and psychiatric diseases such as MDD and discusses the novel therapeutic interventions targeting the KP. The second highest co-cited reference was an article published by Raison et al. (2010), in which the authors found that the activation of IDO induced by peripheral administration of interferon-alpha in patients with hepatitis C conforms with cytokine responses in the brain, thus resulting in increased KYN and QUIN in cerebrospinal fluid, along with increased depressive symptoms. The next two highly co-cited references both belonged to cluster #2, “indoleamine 2,3-dioxygenase.” One was an article published by O'Connor et al. (2009b), which reported that IDO is a critical molecular mediating the LPS-induced depressive-like behavior in mice, probably through an increase in degradation of TRY along an inflammatory pathway (i.e., the KP). The other was a review published by Dantzer et al. (2008), focusing on how peripheral inflammation acts on the brain and results in sickness behavior, and suggesting that pro-inflammatory cytokines may trigger the development of depression via multiple underlying molecular mechanisms, including activation of IDO.

Notably, the most recent research focus was in cluster #9, “gut-brain axis” (mean year 2015), thereby indicating that in the field of the KP in mood disorders, gut microbiota research was still in an early stage. The first representative reference with the strongest currently ongoing citation bursts in this cluster was a review published by O'Mahony et al. (2015) evaluating the evidence of the influence of the gut microbiota on TRY metabolism and the serotonergic system, and exploring the potential mechanisms, including direct and indirect microbial regulation of TRY utilization and 5-HT biosynthesis. The second highest co-cited reference in this cluster focused in detail on the microbial regulation of KP metabolism and reviewed the critical points of gut microbiota control in KP metabolism in both pharmacokinetic and pharmacodynamic aspects (Kennedy et al., 2017). A review published by Foster and McVey Neufeld (2013) had the third highest number of co-citations; in this review, the authors discuss the relationship between stress and microbiota, and how the altered microbiota affect stress-related disorders, including anxiety and depression, thus improving understanding of the microbiota-gut-brain axis. The next representative references in this cluster were two articles: one was published by Jiang et al. (2015), in which the authors found altered fecal microbiota composition in patients with MDD, observed as increased levels of *Enterobacteriaceae* and *Alistipes*, but decreased levels of *Faecalibacterium*; the other was published by Kelly et al. (2016), wherein depression was found to be characterized by reduced richness and diversity in the gut microbiota, and depressed behavior, as well as the alterations in TRY metabolism, were reproduced via fecal microbiota transplantation technology in which the microbiota from depressed patients were transferred to microbiota-depleted rats. These representative references with the highest co-citations in this cluster reflected the important knowledge foundations of gut microbiota research in the field of the KP in mood disorders.

Among the hot topics, the main focus was on QUIN, emerging in 2006, according to the keyword co-occurrence analysis, and the three most relevant topics belonged to the red cluster: IDO, the KP, and major depression. A recent meta-analysis has suggested that KYNA and the KYNA:QUIN ratio decrease, and KYN is preferentially metabolized to the potentially neurotoxic QUIN instead of the neuroprotective KYNA in mood disorders (Marx et al., 2020). Thus, QUIN has been proposed as a potential target and biomarker for mood disorders; e.g., to evaluate the antidepressant effect of ketamine (Verdonk et al., 2019). Among the emerging topics in this field, an overlap analysis identified Alzheimer’s disease (AD), the prefrontal cortex (PFC), and acid as research frontiers, which were in the blue cluster within the keyword co-occurrence network, together with the topics cerebrospinal fluid and BD in this cluster.

AD, one of the most common neurodegenerative disorders, is characterized by progressive memory and mental function loss along with neuropsychiatric symptoms such as depression and anxiety (Erkkinen et al., 2018); it has a prevalence of 10–30% in people over 65 years of age (Eratne et al., 2018). Growing evidence suggests that the KP may play a crucial role in the development of neuropsychiatric symptoms in AD in response to neuroinflammation (Maddison and Giorgini, 2015). A study by Souza et al. (2016) has demonstrated that the elevated pro-inflammatory cytokines lead to the elevated IDO activity, thus subsequently increasing KYN production and the KYN:TRY ratio, whereas decreasing neurotrophic factors in the PFC and hippocampus contribute to the amyloid-beta 1–42-induced neuroinflammation and behavioral abnormalities in mice, thus strongly suggesting a critical role of IDO in mediating the emotional disturbances in AD. Future research may focus on the mutual effects of the gut microbiota on AD and KP rate-limiting enzymes such as IDO (Dehhaghi et al., 2019). Recent studies have proposed perspectives and the potential role of gut microbiota modulation in AD (Garcez et al., 2019); for example, gut microbiota-derived vitamins may be used as possible interventions for psychiatric treatment in AD (Rudzki et al., 2021).

The PFC was another emerging topic identified with the strongest citation bursts. A previous study by Liu et al. (2017) has suggested imbalanced KP metabolism in the PFC, as reflected by a decrease in prefrontal KYNA in Flinders Sensitive Line rats, which might possibly be associated with the induction of the KP enzymes by pro-inflammatory cytokines. However, a study by Clark et al. (2016) has indicated that depression is associated with unexpectedly decreased KP metabolism and cytokine expression in the ventrolateral PFC, which is part of the orbitofrontal cortex region associated with higher emotional function, thus indicating that the brain KP regulation may be region-specific. A recent study has shown that IDO1 expression in mice is upregulated by LPS in the PFC but not in the hippocampus, and microinjection of 1-MT (a potent IDO1 antagonist) or microRNA-874-3p into the PFC downregulates LPS-induced IDO1 expression and ameliorates LPS-induced depression-like behavior, thus revealing that microRNA-874-3p is a novel potential target for the treatment of MDD (Suento et al., 2020). Future research may examine the influence of the gut microbiota on enzymes involved in TRY metabolism along the KP in PFC. For example, a recent study by Xie et al. (2020) has reported that oral treatment with *Lactobacillus reuteri* 3 has anti-depressive effects through increasing the expression of enzymes involved in 5-HT biosynthesis, but inhibiting that of the KP enzymes, including IDO in the colon and PFC in mice with depression-like symptoms induced by chronic social defeat stress.

### Limitations

Despite following certain bibliometric principles and comprehensive analysis strategies, our present study has some inevitable limitations. First, only English articles and reviews published within a particular period of time from the WoSCC and Scopus databases were used, thus potentially leading to language and publication biases. For example, although the retrieval time scope was sufficiently long to reflect the research trends in our field of focus, some critical, seminal original papers that gave rise to this field may be missing. These papers, such as those first describing the compounds involved in the KP, formed the basis of the research of the authors cited; thus, historical background information may need to be added in these types of bibliometric analysis. Additionally, in some cases, the identified researchers might not have published original discoveries but instead published large numbers of reviews, which were then cited frequently. This is a well-recognized anomaly in the citation analysis system that may yield misleading results regarding the contributions to the field. Second, only some specific terms referring to the main KP metabolites were included in our retrieval strategy, and consequently, the publications retrieved may contain possible false positives and false negatives, given that no search query is 100% perfect (Sweileh et al., 2017). Third, to date, there is a lack of adherence to internationally accepted ethical standards in bibliometric analysis, and the limitations of analytic tools may provide a subjective view of individual work and contributions. Therefore, we emphasize that the information generated and discussed has no relationship to the nature, originality or importance of the work, because it includes information consisting largely, or sometimes entirely, of reviews of original work by other people. Despite these limitations, given the sufficiently large number of collected documents in the present analysis, we believe that our findings provide a more comprehensive picture of research on the KP in mood disorders, especially involving gut microbiota research, which may help provide an instructive perspective on the current research and direct future research in this field.

## Data Availability

The raw data supporting the conclusions of this article will be made available by the authors, without undue reservation.

## Conclusion

This is the first bibliometric analysis to comprehensively evaluate the general aspects of, and future trends in, the field of the KP in mood disorders, particularly the gut microbiota research in this field. Overall, we found rapid growth in publications in this field since 2006, which was generally favored by high-IF journals in the field of neuropsychiatry. More widespread global collaboration may be necessary, particularly for China. The most important and most recent research focuses were the “kynurenine pathway” and “gut-brain axis,” respectively, reflecting the research foundations in this field. Journal co-citation analysis showed a strong tendency toward co-citation of research in the field of psychiatry. Reference co-citation analysis revealed the key role of the KP in bridging the gut microbiota and the host immune system, and gut microbiota research has become the most recent focal point in the field of the KP in mood disorders. The largest research hotspot has become “quinolinic acid,” appearing in 2006. Future research should focus on the topics of “Alzheimer’s disease,” “prefrontal cortex,” and “acid,” which were identified as research frontiers in this field. These timely analysis results provide a new glimpse into research on the KP in mood disorders, particularly focusing on the microbiota-gut-brain axis in this field, and may help researchers choose suitable journals for publication, find potential collaborators, and understand hotspots and frontiers, thus promoting the development of this field.
